# Exosome‐derived ANXA9 functions as an oncogene in breast cancer

**DOI:** 10.1002/cjp2.334

**Published:** 2023-06-09

**Authors:** Cuiping Lu, Ying Zhan, Yunshan Jiang, Jianrong Liao, Zidan Qiu

**Affiliations:** ^1^ Department of Medical Oncology Longyan First Affiliated Hospital of Fujian Medical University Longyan Fujian PR China

**Keywords:** breast cancer, *ANXA9*, exosomes, oncogene, proliferation

## Abstract

Breast cancer (BCA) is one of the most prevalent cancers among women. Emerging evidence has revealed that Annexin A‐9 (*ANXA9*) plays a crucial function in the development of some cancers. Notably, *ANXA9* has been reported to be a new prognostic biomarker for gastric and colorectal cancers. However, its expression and biological function in BCA have not yet been investigated. Using online bioinformatics tools such as TIMER, GEPIA, HPA, and UALCAN, we predicted *ANXA9* expression and its correlation with the clinicopathological characteristics of BCA patients. RT‐qPCR and western blot were utilized to measure *ANXA9* mRNA and ANXA9 protein expression in BCA patient tissues and cells. BCA‐derived exosomes were identified by transmission electron microscopy. Functional assays were employed to evaluate the biological role of *ANXA9* in BCA cell proliferation, migration, invasion, and apoptosis. A tumor xenograft *in vivo* model was utilized to assess the role of *ANXA9* in tumor growth in mice. Bioinformatics and functional screening analysis revealed that *ANXA9* was highly expressed in BCA patient tissues, with median *ANXA9* expression 1.5‐ to 2‐fold higher than in normal tissues (*p* < 0.05). RT‐qPCR confirmed that *ANXA9* expression in BCA tissues was around 1.5‐fold higher than the adjacent normal tissues (*p* < 0.001). *ANXA9* expression in different subtypes of BCA also showed a difference, and *ANXA9* was found to be mostly significantly upregulated in luminal BCA relative to normal tissues or other histological subtypes (*p* < 0.001). Moreover, *ANXA9* expression was elevated in different races, ages, clinical stages, node metastasis status, and menopause status groups relative to the normal group (*p* < 0.001). Furthermore, *ANXA9* was found to be secreted by BCA tissue‐derived exosomes and its expression was upregulated 1‐ to 7‐fold in BCA cells treated with exosomes (*p* < 0.001), while its expression in MCF10A cells was not significantly altered by treatment with exosomes (*p* > 0.05). *ANXA9* silencing induced a significant decrease of around 30% in the colony number of BCA cells (*p* < 0.01). The number of migrated and invaded BCA cells also decreased by around 65 and 68%, respectively, after silencing *ANXA9* (*p* < 0.01). Tumor size was significantly reduced (nearly half) in the LV‐sh‐*ANXA9* group relative to the LV‐NC group in the xenograft model (*p* < 0.01), suggesting that *ANXA9* silencing repressed tumor progression in BCA progression *in vitro* and *in vivo*. In conclusion, exosome‐derived *ANXA9* functions as an oncogene that facilitates the proliferation, migration, and invasiveness of BCA cells and enhances tumor growth in BCA development, which may provide a new prognostic and therapeutic biomarker for BCA patients.

## Introduction

Breast cancer (BCA) is one of the most common cancer types and accounts for 15% of cancer‐related death in women globally [1]. It is a heterogeneous disease with diverse subtypes [2]. The 5‐year survival rate is approximately 90% for patients in the early stage of this disease [3] but less than 40% for patients with late‐stage disease [4]. At present, the known conventional treatments include surgery, radiotherapy, chemotherapy, and immunotherapy [5]. Among all BCA patients with metastatic disease, the prognosis is worst in patients with triple‐negative breast cancer (TNBC) [6]. It has been confirmed that metastasis is the main cause of BCA‐related death, which greatly hinders the success of treatment [7]. Therefore, a more comprehensive understanding of the underlying mechanism of BCA metastasis is particularly important for improving the prognosis and treatment of BCA patients.

Annexins are a class of Ca^2+^‐dependent phospholipid‐binding proteins, which can be classified into five subgroups (A, B, C, D, and E) [8]. The Annexin A (ANXA) group has 13 members (*ANXA1*–*ANXA13*), which exist in different human organs [9]. Particularly, a majority of the members of the ANXA group are confirmed to be associated with tumorigenesis and development of different cancer types [10]. For example, *ANXA1* depletion can abrogate immune repression in TNBC [11]. *ANXA1* upregulation accelerates cell invasive capability in prostate cancer [12]. *ANXA2* upregulation facilitates esophageal squamous cell carcinoma cell migration and invasion through activating the MYC‐HIF1A‐VEGF cascade [13]. High expression of *ANXA3* is an independent prognostic indicator in gastric cancer and its knockdown can inhibit tumor growth [14]. *ANXA9* belongs to the ANXA group and its regulatory role has also been explored in some cancer types [15]. It has been reported that *ANXA9* promotes metastasis of colorectal cancer and predicts poor prognosis [16]. *ANXA9* has been shown to be highly expressed in gastric cancer tissues and proposed as a new prognostic biomarker associated with immune infiltration in gastric cancer [17]. Importantly, a recent bioinformatic study suggested that *ANXA9* might be associated with BCA prognosis and might act as a potential prognostic indicator [18]. However, there is no direct evidence exploring the expression and functional role of *ANXA9* in BCA.

The main purpose of this study was to investigate the expression and functional role of *ANXA9* in BCA. We utilized different bioinformatics tools and functional assays to analyze the biological function of *ANXA9* in BCA.

## Materials and methods

### Bioinformatics analysis

The pan‐cancer expression profile of *ANXA9* was analyzed using the TIMER database (http://timer.cistrome.org/), which is a resource for immune infiltrates and differential gene expression analysis between tumor and normal tissues [19]. The expression of *ANXA9* in BCA/normal samples or in different BCA stages (stages I–X) was analyzed using the Gene Expression Profiling Interactive Analysis (GEPIA) database, which is a valuable resource to analyze gene expression patterns in different tumors or in different cancer pathological stages based on tumor and normal samples from the TCGA and the GTEx databases [20]. Gene expression correlation analysis in BCA was also performed using Pearson correlation analysis based on the GEPIA database. The subcellular location and expression of ANXA9 in different cell types were analyzed using the Human Protein Atlas (HPA) database (https://www.proteinatlas.org/) [21], which provides access to protein expression profiles in different tissues and cells as well as the cellular distribution information. The *ANXA9* expression pattern in BCA patients based on clinicopathologic factors was analyzed using the UALCAN database (http://ualcan.path.uab.edu/index.html) [22].

### Protein–protein interaction network construction

The protein–protein interaction (PPI) network of the interaction of ANXA9 and other associated proteins, and the functional enrichment analysis (cellular component) of ANXA9 were analyzed using the Search Tool for the Retrieval of Interacting Genes (STRING) database (https://cn.string-db.org/) [23].

### Patient tissue samples

A total of 30 surgically resected BCA tissues and adjacent noncancerous tissues (termed para‐carcinoma tissues) were examined in the present study. All patients underwent surgical resection at Longyan First Affiliated Hospital of Fujian Medical University, between 2018 and 2020. None of the patients received radiotherapy or chemotherapy before surgery. This study was approved by the Ethics Committee of Longyan First Affiliated Hospital of Fujian Medical University. Written informed consent was acquired from all patients. After resection, the samples were frozen and stored at −80 °C.

### Cell culture and transfection

BCA cell lines MCF7, SK‐BR‐3, and T‐47D cells and normal human mammary epithelial cell line MCF10A were purchased from ATCC (Manassas, VA, USA). The cells were cultured in RPMI‐1640 media (Gibco, Pacheco, CA, USA) with 10% FBS at 37 °C with 5% CO_2_.

To silence *ANXA9* expression, MCF7 and T‐47D cells were transfected with 25 nm of *ANXA9* shRNA (sh‐*ANXA9*; Genechem, Shanghai, PR China, sequences: CCGG‐GCAGCTCATCTCACGAAACTT‐CTCGAG‐AAGTTTCGTGAGATGAGCTGC‐TTTTTG) and the negative control shRNA (sh‐NC; Genechem, sequences: CCGG‐ATCATTAGGAAGTGGTCGAAG‐CTCGAG‐CTTCGACCACTTCCTAATGAT‐TTTTTG) utilizing Lipofectamine 3000 (Invitrogen, CA, USA) for 48 h. In order to establish stable knockdown in MCF7 and T‐47D cells, lentiviruses (LVs; Genechem) were utilized as the vector to carry the shRNA sequences and then transfected into cells.

### Reverse transcription‐quantitative polymerase chain reaction

Total RNA was subjected to extraction by utilizing TRIzol (Invitrogen). The cDNA templates were synthesized by a Transcriptor First Strand cDNA Synthesis kit (Takara, Shiga, Japan). Next, qPCR utilizing SYBR Green II (Takara) was conducted with an ABI PRISM 7900 Sequence Detector system (Applied Biosystems, Waltham, MA, USA). *ANXA9* expression was measured by 2^−ΔΔCt^ method and normalized to *GAPDH*. The primer sequences used in this study were:

*ANXA9*: forward: 5′‐TACTGAGGGCCATTACTGG‐3′, reverse: 5′‐TTCATCAGGTCCTGTTGGG‐3′

*GAPDH*: forward: 5′‐TCAAGATCATCAGCAATGCC‐3′, reverse: 5′‐CGATACCAAAGTTGTCATGGA‐3′

### Western blot

Cells were lysed with RIPA buffer (Thermo Fisher Scientific, Waltham, MA, USA), followed by measuring the proteins with the bicinchoninic acid kit (Beyotime, Shanghai, PR China). Twenty micrograms protein per sample were electrophorized with 10% SDS‐PAGE and transferred onto PVDF membranes (Millipore, Shanghai, PR China). After blocking the membranes with 5% skim milk, membranes were incubated with primary antibodies against ANXA9 (cat. no. ab166621, Abcam, Cambridge, MA, USA), CD63 (cat. no. ab231975, Abcam), CD81 (cat. no. ab109201, Abcam), CD9 (cat. no. 223052, Abcam), TSG101 (cat. no. ab228013, Abcam), and GAPDH (cat. no. ab9485, Abcam) at 4 °C overnight. Next, membranes were incubated with the secondary antibody for 2 h. Protein bands were detected by using enhanced chemiluminescence (Advansta, Menlo Park, CA, USA). The data were analyzed by ImageJ software (National Institutes of Health, Bethesda, MD, USA).

### Colony formation assay

The transfected cells were inoculated into six‐well plates (1,000 cells/well) and cultured in an incubator at 37 °C. The culture medium was changed every third day. After 2 weeks of culture, cells were subjected to fixation with 4% paraformaldehyde for 30 min. Then, cells were stained with crystal violet dye for 15 min. The cell colonies were counted under a Stereo microscope (Boston Industries, Walpole, MA, USA).

### Transwell assays

The transfected cells (1 × 10^5^) were suspended in serum‐free medium and placed in the upper transwell chambers (Corning, New York, NY, USA) coated with or without Matrigel for performing invasion or migration assays. The lower chamber was supplemented with media containing 10% FBS. After 24 h of incubation, the migrated cells in the lower chamber were fixed in 4% paraformaldehyde and stained with crystal violet for 15 min. The number of migrated and invaded cells was photographed and recorded under a microscope (Olympus, Tokyo, Japan) and counted using ImageJ software.

### Flow cytometry

Apoptosis in BCA cells was detected using the Annexin V‐FITC/PI detection kit (BD Biosciences, San Diego, CA, USA). In brief, the transfected cells were rinsed with PBS and then re‐suspended in binding buffer comprising 5 μl Annexin V‐FITC and 5 μl PI. Cells were incubated for 10 min in the dark. A flow cytometer (BD Accuri C6, Boston Industries) was employed for acquiring the cells, while the apoptosis rate was measured by using Flow Jo software.

### Isolation of exosomes

Tumor tissues were cut into 1 cm^3^ pieces and inoculated into serum‐free culture medium. After 24 h, supernatant was collected and then subjected to centrifugation at 300 × *g* for 10 min, followed by filtering through a 0.22‐μm pore filter (Millipore). Then, the exosome quick extraction solution (System Biosciences, Palo Alto, CA, USA) was added and mixed by inverting the tubes several times. After being incubated at 4 °C overnight, the mixture was centrifuged at 1,500 × *g* for 30 min at room temperature to recover the exosomes. Exosomes particles were visualized with a Hitachi 7100 transmission electron microscope (Hitachi, Tokyo, Japan).

### Proteinase and RNase assays

After isolation, exosomes were collected and either treated with 0.1% Triton X‐100 (Sigma‐Aldrich, Santa Clara, CA, USA) or cultured with RNase A (Solarbio, Beijing, PR China) for 20 min or with proteinase K (Solarbio) for 10 min. After that, Proteinase K was inactivated by mixing with PMSF at room temperature for 10 min and at 90 °C for 5 min. Next, RNase A was supplemented into the mixture. RNA was then extracted from exosomes and assessed through RT‐qPCR or western blot to evaluate the expression of *ANXA9*.

### Tumor xenograft *in vivo* model

The animal experiments were approved by our hospital's ethics committee for the care and use of animals (approval number: NS2019U‐003A). A total of 30 BALB/c nude mice (3–4 weeks old) were purchased from Vital River Center (Beijing, PR China) and raised in a pathogen‐free environment. Mice were separated into LV‐NC and LV‐sh‐*ANXA9* groups (*n* = 15 in each group). MCF7 cells (1 × 10^7^) transfected with LV‐NC or LV‐sh‐*ANXA9* were subcutaneously injected into the right rear flank of mice. After 4 weeks, mice were euthanized, and tumor samples were obtained and photographed; tumor volume and weight were also measured. The tumor volume (mm^3^) was calculated as: tumor volume = length × width^2^ × 0.5.

### Immunohistochemistry

After mice were euthanized, the tumor tissues were fixed in 10% formalin, embedded in paraffin, and cut into 4‐μm‐thick sections. Next, the tissue sections were incubated with anti‐Ki‐67 antibody (1:100; cat. no. ab15580, Abcam) overnight at 4 °C and then incubated with HRP‐conjugated secondary antibody for 2 h. Finally, the sections were counterstained utilizing a DAB kit (Abcam) and then observed through microscopy.

### TUNEL assay

The paraffin‐embedded tissue sections were dewaxed and then fixed with 4% paraformaldehyde for half an hour. Then, the sections were incubated with 0.3% Triton X‐100 on ice for 2 min. Later, TUNEL solution was prepared based on the user guides of TUNEL detection kit (Beyotime) and then tissue sections were cultured with TUNEL reaction mixture. A fluorescence microscope (Olympus) was used for analysis.

### Statistical analyses

GraphPad Prism 8 software was utilized for statistical analysis. Data were displayed as the means ± SD from three individual repeats. The Wilcoxon test was utilized to compare the association between *ANXA9* and clinicopathological characteristics in BCA patients. The *t* test or one‐way analysis of variance (ANOVA) followed by Tukey's *post hoc* test was utilized to evaluate the differences between groups. *P* < 0.05 represented statistical significance.

## Results

### *ANXA9* expression in BCA

**Figure 1:**
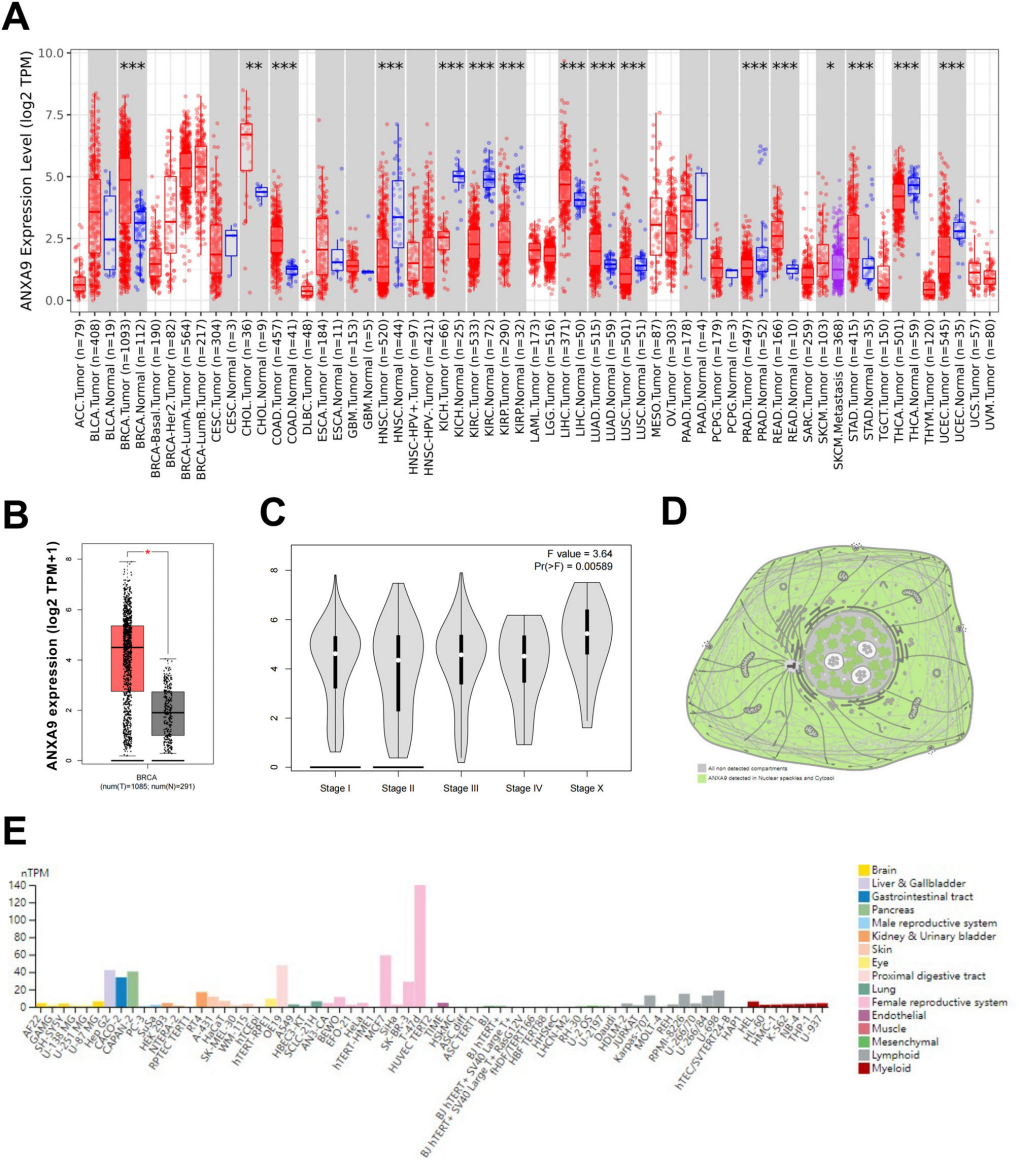
The expression of *ANXA9* in BCA. (A) *ANXA9* expression in different cancer types was obtained from the TIMER database. (B, C) The expression of *ANXA9* in BCA and normal tissues and at different stages of BCA was predicted using the GEPIA database. (D, E) ANXA9 subcellular location and expression in different organs were obtained from the HPA database. Significance: *p < 0.05; **p < 0.01; ***p < 0.001.

To investigate the differences in *ANXA9* expression between tumor and normal samples, the *ANXA9* expression levels in various cancer types were examined using the TIMER database (http://timer.cistrome.org/). *ANXA9* was strongly expressed in invasive breast carcinoma, cholangiocarcinoma, colon adenocarcinoma, liver hepatocellular carcinoma, lung adenocarcinoma, lung squamous cell carcinoma, rectum adenocarcinoma, skin cutaneous melanoma, and stomach adenocarcinoma when compared to normal tissues (Figure 1A; *p* < 0.05). Interestingly, we also noticed low *ANXA9* expression in various cancers (Figure 1A; *p* ˃ 0.05). To further verify the *ANXA9* expression levels in BCA and normal tissues, the GEPIA database (http://gepia2.cancer-pku.cn/#index) was utilized. *ANXA9* was highly expressed in BCA tumor tissues compared with normal tissues (Figure 1B; *p* < 0.05). The expression of *ANXA9* in different BCA stages was compared on the GEPIA database using one‐way ANOVA. *ANXA9* was identified to be differentially expressed in various BCA stages as shown in Figure 1C (*F* value = 3.64, *p* < 0.05). Next, the HPA database (https://www.proteinatlas.org/) was utilized to predict the subcellular localization of ANXA9. ANXA9 was mainly localized to nuclear speckles and the cytosol (Figure 1D). Furthermore, this database was also utilized to detect the expression of ANXA9 in different organs, and we observed that ANXA9 was most prominently overexpressed in female reproductive system‐related cell lines (pink bars) (Figure 1E). Collectively, these results suggest that *ANXA9* is highly expressed in BCA tissues, and its dysregulation may be associated with the progression of BCA.

### Relationship of *ANXA9* expression with different clinicopathological parameters in BCA patients

**Figure 2:**
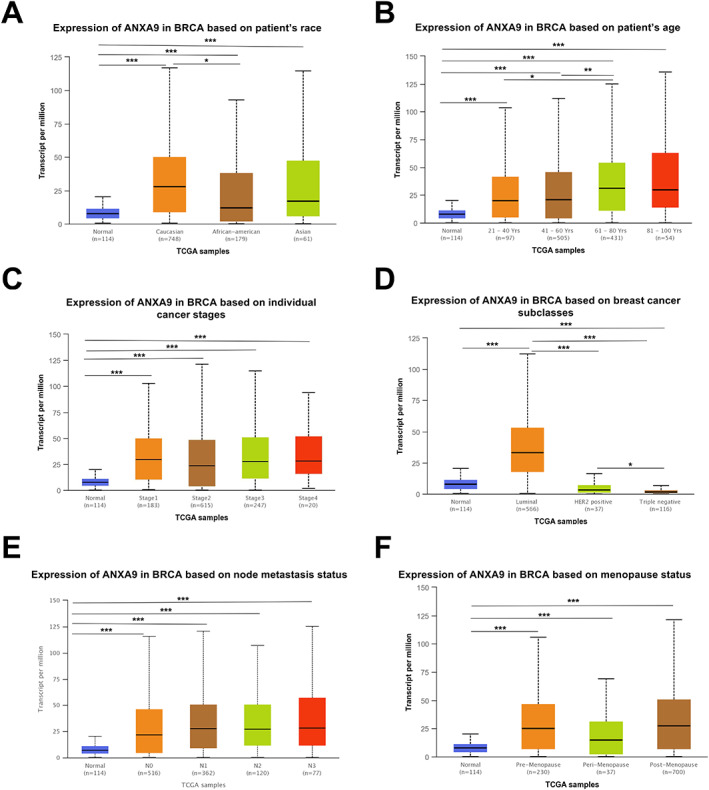
Relationship between *ANXA9* expression and clinicopathological characteristics of BCA patients. (A–F) *ANXA9* mRNA expression levels in different BCA patients based on clinical parameters (race, age, cancer stage, subclasses, metastasis, and menopause status) were obtained from the UALCAN database. Significance: *p < 0.05; **p < 0.01; ***p < 0.001.

As we had demonstrated that *ANXA9* was highly expressed in BCA patients (Figure 1), we then investigated the correlation between *ANXA9* expression and clinicopathological traits in BCA patients. The UALCAN database (http://ualcan.path.uab.edu/index.html) revealed that *ANXA9* levels were elevated in tumor tissues of Caucasian, African‐American, and Asian BCA patients compared to the normal group (Figure 2A; *p* < 0.05). Among these, *ANXA9* expression in tumor tissues of Caucasian BCA patients was the highest, followed by Asian BCA patients, and then the African American BCA patients (Figure 2A). In terms of age distribution, *ANXA9* remained highly expressed in BCA patients of different ages (21–40, 41–60, 61–80, and 81–100 years). *ANXA9* expression in tumor tissues of BCA patients aged 61–80 years was markedly higher than patients aged 21–40 or 41–60 years (*p* < 0.05), suggesting *ANXA9* expression gradually increased with the age of BCA patients (Figure 2B). Additionally, in terms of pathologic staging, *ANXA9* was found to be highly expressed in BCA patients in stages I, II, III, and IV (Figure 2C; *p* < 0.05). Furthermore, *ANXA9* upregulation was observed in luminal BCA patients; however, *ANXA9* downregulation was observed in HER2‐positive BCA and TNBC patients compared to normal controls (Figure 2D). Subsequently, based on node metastasis status, *ANXA9* expression was higher in BCA patients classified as N0, N1, N2, or N3 (Figure 2E; *p* < 0.05). Moreover, *ANXA9* overexpression was also observed in BCA patients in pre‐menopause, perimenopause, and post‐menopause (Figure 2F; *p* < 0.05). Taken together, these data indicate that *ANXA9* is highly expressed in different races, ages, cancer stages, node metastasis stages, and menopause status of BCA patients relative to the normal group. In addition, we also found that *ANXA9* is upregulated in luminal BCA patients while downregulated in HER2‐positive BCA and TNBC relative to the normal group, suggesting the potential of *ANXA9* in biomarker selection for BCA patients.

### *ANXA9* expression in BCA patient tissues and cell lines

**Figure 3:**
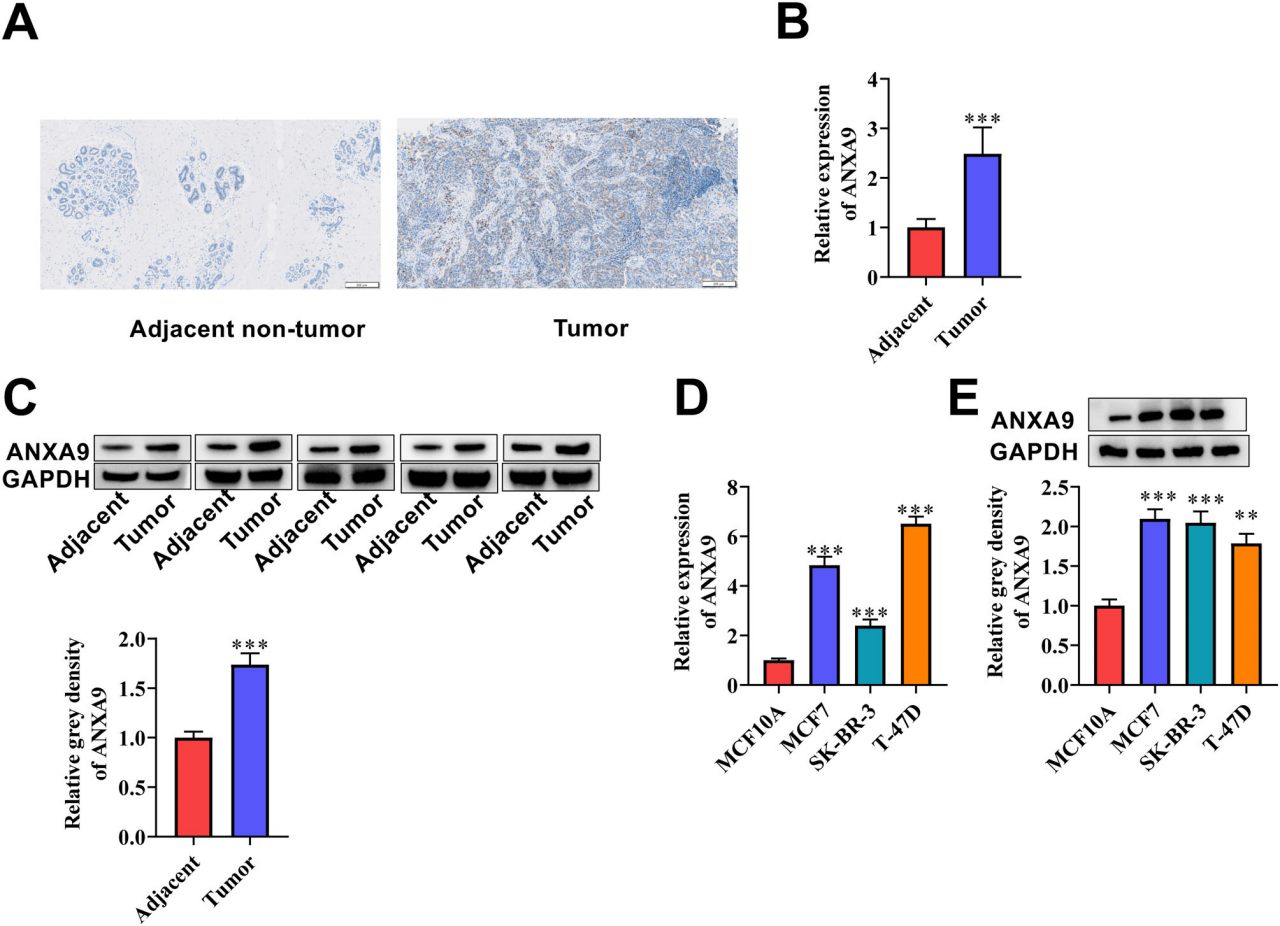
Validation of *ANXA9* expression in BCA. (A) ANXA9 expression in adjacent non‐tumor tissues and BCA tissues was detected by IHC staining. (B) RT‐qPCR assay was to measure *ANXA9* expression in adjacent non‐tumor tissues and BCA tissues in 30 patients. (C) Western blot analysis of ANXA9 protein levels in adjacent non‐tumor and tumor tissues of five patients. GAPDH was used as an internal control. (D) RT‐qPCR and (E) western blot analysis of *ANXA9* mRNA and ANXA9 protein levels in normal MCF10A cells, and BCA cell lines MCF7, SK‐BR‐3, and T‐47D cells. Significance: **p < 0.01; ***p < 0.001.

Since bioinformatic analysis suggested that *ANXA9* is highly expressed in BCA patients, therefore, we next validated ANXA9 protein expression in BCA patient tissues and a series of BCA cell lines. Immunohistochemistry (IHC) staining revealed that ANXA9 expression was high in tumor tissues compared to the adjacent nontumor tissues (Figure 3A). We further confirmed ANXA9 expression via RT‐qPCR and western blot analysis. ANXA9 mRNA and protein levels were upregulated in tumor tissues in contrast to adjacent non‐tumor tissues (Figure 3B,C; *p* < 0.05). These results were further validated in BCA cell lines where ANXA9 was also overexpressed in MCF7, SK‐BR‐3, and T‐47D cells compared with the normal MCF10A cells (Figure 3D,E; *p* < 0.05). These results confirm the bioinformatics data that ANXA9 is indeed highly expressed in BCA patients. Since ANXA9 was highly expressed in MCF7 and T‐47D cells, we therefore used these two cell lines for further assays.

### ANXA9 is associated with exosomes

**Figure 4:**
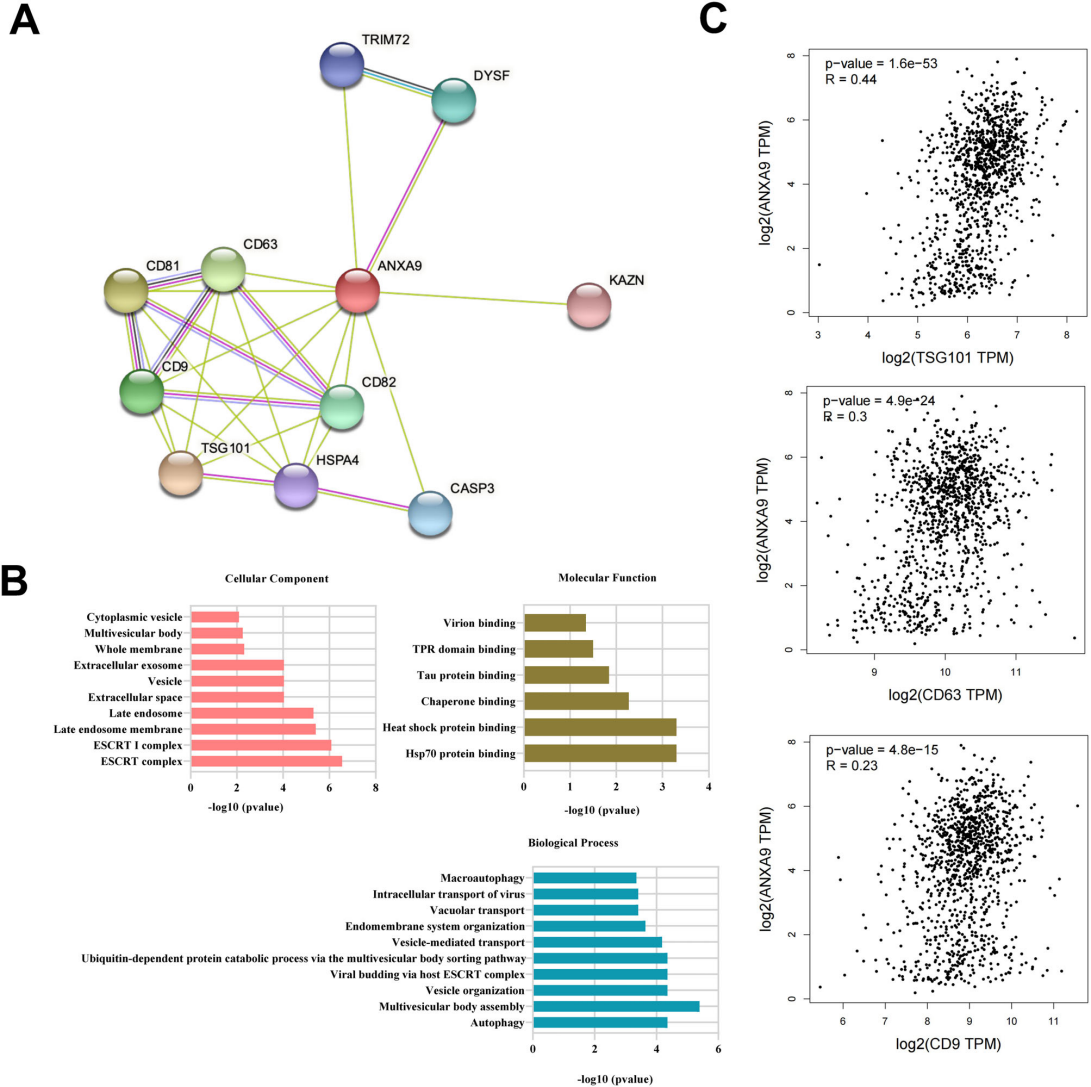
Functional enrichment analysis of ANXA9. (A, B) The PPI network and GO functional enrichment analysis of ANXA9 were predicted by STRING. (C) The correlation of *ANXA9* and *TSG101*/*CD63*/*CD9* in BCA tissues was predicted by GEPIA.

Next, we utilized the STRING database (https://cn.string-db.org/) to generate the PPI network of ANXA9. According to this network, we found that the exosome markers CD81, CD9, CD63, and TSG101 were associated with ANXA9, suggesting that ANXA9 may exist in exosomes in tumor tissues (Figure 4A). Functional enrichment analysis further confirmed this conjecture that ANXA9 was enriched in terms such as extracellular exosome, extracellular space, cytoplasmic vesicle, and vacuolar transport based on gene ontology (GO) analysis on the data from the STRING database (Figure 4B). Moreover, the GEPIA database showed that *ANXA9* expression was notably correlated with TSG101, CD9, and CD63 levels in BCA (Figure 4C). These results suggest the possible association of ANXA9 with exosomes.

### *ANXA9* is secreted by BCA tissue‐derived exosomes

**Figure 5:**
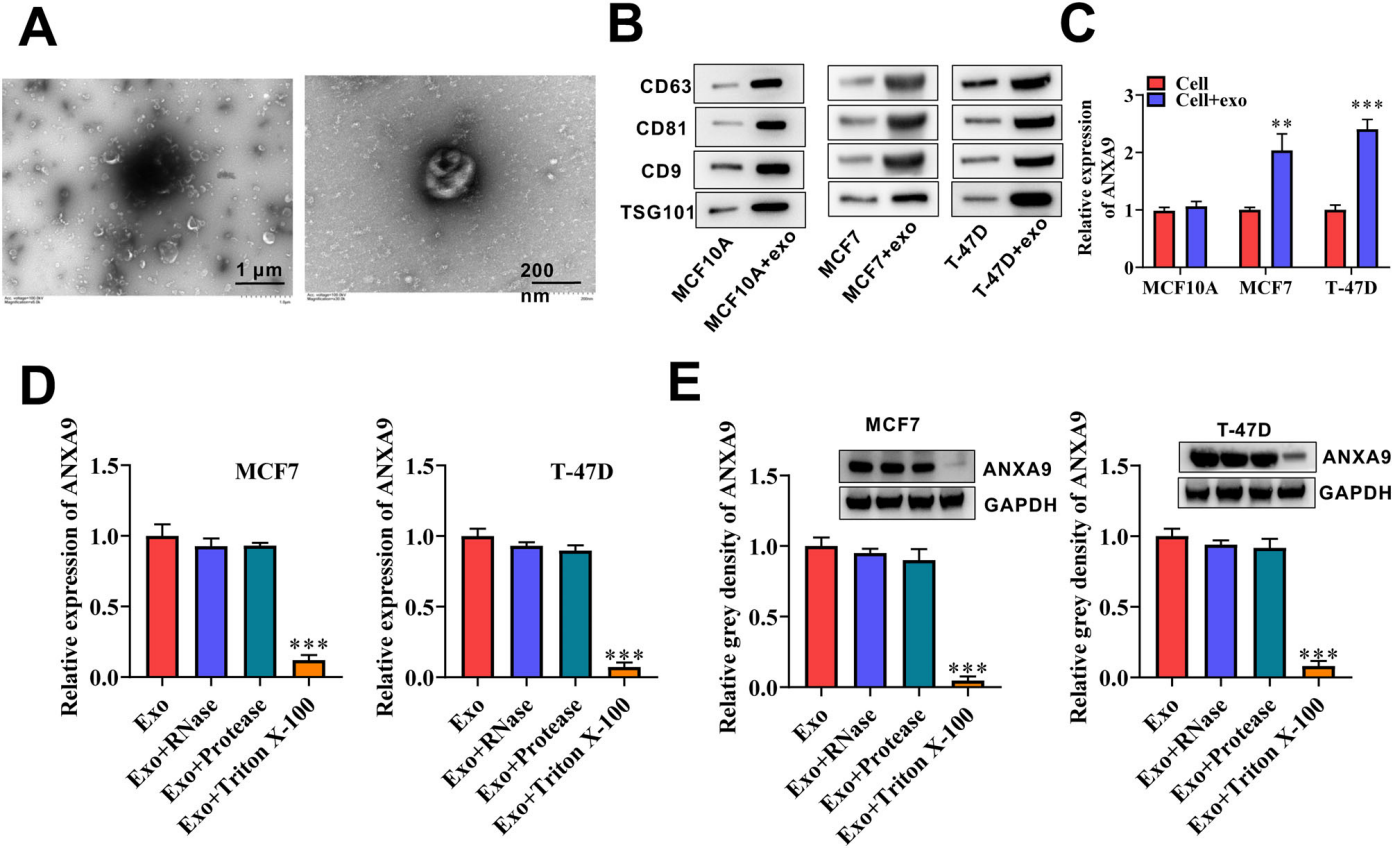
*ANXA9* is secreted by BCA tissue‐derived exosomes. (A) Exosomes at different magnifications by transmission electron microscope. (B) Western blot was utilized to detect exosomal markers CD63, CD81, and CD9 protein levels in MCF10A, MCF, and T‐47D cells cultured with or without exosomes. (C) *ANXA9* mRNA expression level in MCF10A, MCF7, and T‐47D cells cultured with or without exosomes was tested by RT‐qPCR. (D, E) Proteinase and RNase protection assays were utilized to test *ANXA9* mRNA and ANXA9 protein expression levels in MCF7 and T‐47D cells cultured with exosomes and treated with/without RNase/Protease/Triton X‐100. Significance: **p < 0.01; ***p < 0.001.

In order to clarify whether *ANXA9* is secreted by BCA tissue‐derived exosomes, we isolated exosomes from BCA tissues and verified them by electron microscopy (Figure 5A). We then detected the protein expression levels of exosomal markers such as CD63, CD81, CD9, and TSG101 in MCF10A, MCF7, and T‐47D cells cultured with or without exosomes. Western bolt results illustrated that the cells cultured with exosomes had high protein expression levels of exosomal markers CD63, CD81, CD9, and TSG101 in comparison to the cells cultured without exosomes (Figure 5B). Next, we evaluated the mRNA expression level of *ANXA9* in MCF10A, MCF7, and T‐47D cells cultured with or without exosomes. Interestingly, BCA cells cultured with exosomes had significantly higher expression of *ANXA9* compared to the cells cultured without exosomes. However, *ANXA9* showed no significant difference in MCF10A cells cultured with or without exosomes, which indicated that the effects of exosomes on ANXA9 content were specific to cancer cells (Figure 5C; *p* < 0.05). Moreover, the outcome of RT‐qPCR and western blot indicated that ANXA9 resided in the exosome lumen and was protected against RNase (Figure 5D,E). Taken together, these results indicate that *ANXA9* is secreted by BCA tissue‐derived exosomes.

### *ANXA9* functions as an oncogene in BCA

**Figure 6:**
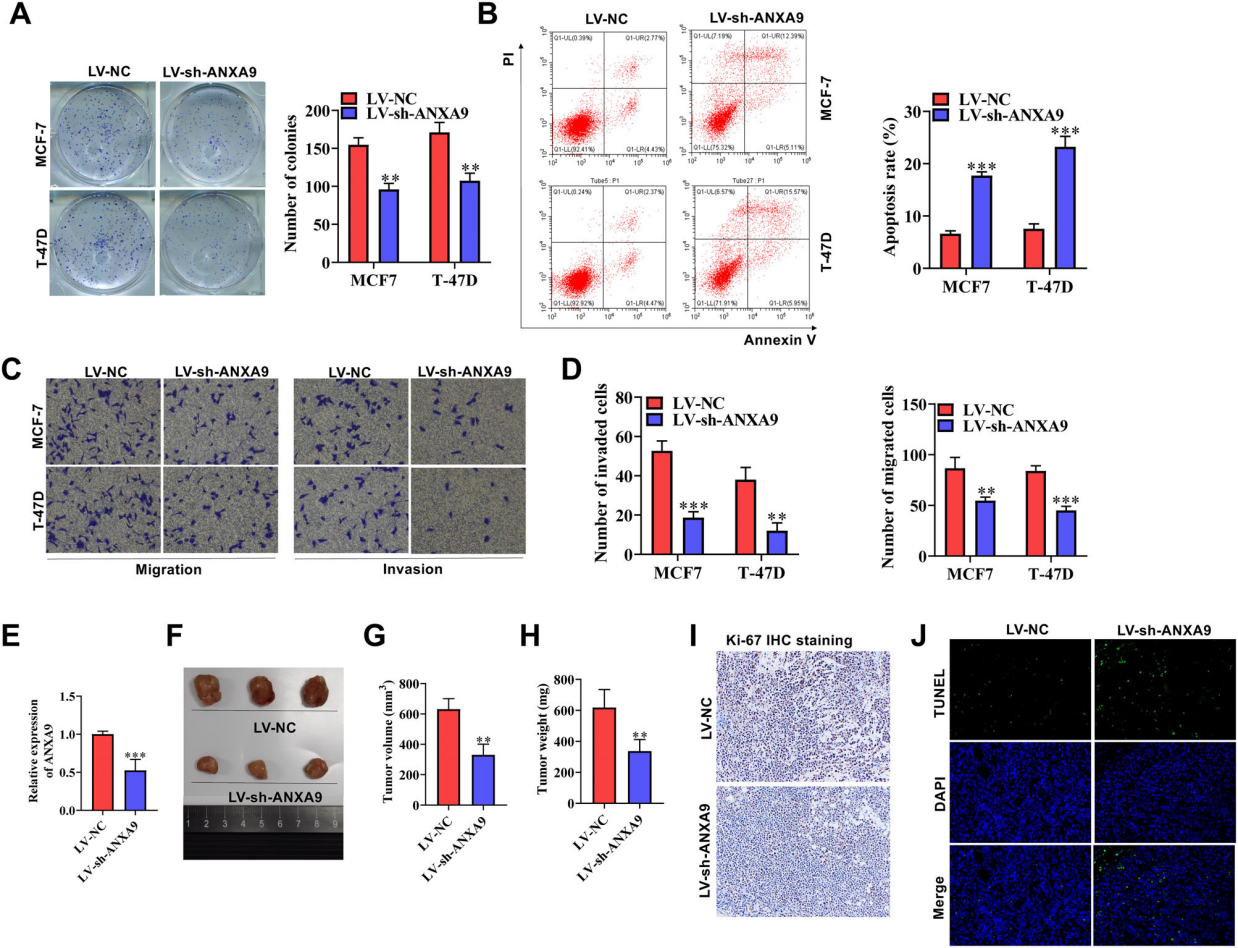
*ANXA9* silencing suppresses BCA progression *in vitro* and *in vivo*. (A) Colony formation assay was applied for estimating cell proliferation when *ANXA9* was silenced. (B) Cell apoptotic capability was determined by flow cytometry analysis when *ANXA9* was knocked down. (C, D) Transwell assay was employed for measuring cell migratory and invasive capabilities when *ANXA9* was knocked down. (E) RT‐qPCR analysis of *ANXA9* expression in tumor tissues of mice injected with MCF7 cells transfected with LV‐NC or LV‐sh*‐ANXA9*. (F, H) Tumor size, volume, and weight in indicated groups. (I) IHC staining assay was performed to measure Ki‐97 expression in mice injected with LV‐NC or LV‐sh‐*ANXA9* transfected MCF7 cells. (J) TUNEL assay was utilized for measuring cell apoptosis in mice injected with LV‐NC or LV‐sh‐*ANXA9* transfected MCF7 cells. Significance: **p < 0.01; ***p < 0.001.

Then *ANXA9* expression was knocked down in MCF7 and T‐47D cells by transfecting with sh‐*ANXA9* LV vector. As shown in Figure 6A, MCF7 and T‐47D cells had increased numbers of colonies in the LV‐NC group; however, when we knocked down *ANXA9*, the number of colonies was significantly reduced in these cells, suggesting cell proliferative capability was suppressed (*p* < 0.05). Flow cytometry analysis illustrated that the cell apoptosis rate was reduced in the LV‐NC group which was elevated by *ANXA9* knockdown (Figure 6B; *p* < 0.05). Furthermore, we conducted transwell assays and observed that *ANXA9* silencing markedly decreased the quantity of migrated cells and invaded cells in comparison to the LV‐NC group in which the numbers of migrated and invaded cells were high (Figure 6C,D; *p* < 0.05). We then established the mouse xenograft tumor model. After inoculation of MCF7 cells transfected with LV‐NC or LV‐sh‐*ANXA9* for 4 weeks, mice were euthanized, and tumor samples were collected. In the tumor‐bearing mouse models, *ANXA9* expression in the LV‐sh‐*ANXA9* group was lower than the control group (Figure 6E; *p* < 0.05). Interestingly, we found that tumor growth was slower in nude mice injected with LV‐sh‐*ANXA9* transfected MCF7 cells than in the control group (Figure 6F; *p* < 0.05). Subsequently, tumor volume and weight were also found to be significantly reduced in mice injected with LV‐sh‐*ANXA9* (Figure 6G,H; *p* < 0.05). Ki‐67 IHC staining showed that the quantity of Ki‐67 positive cells in the LV‐sh‐*ANXA9* group was notably reduced in comparison to the control group (Figure 6I). Furthermore, tumor cell apoptosis was measured *via* TUNEL assay. Compared with the LV‐NC group, cell apoptosis in the LV‐sh‐*ANXA9* group was notably increased (Figure 6J). Overall, *ANXA9* silencing reduces cell proliferative, migratory, and invasive capabilities *in vitro* and reduces BCA tumor progression *in vivo*.

## Discussion

BCA is one of the most common cancer types prevalent among women all over the world [24]. The prognosis of BCA patients is usually poor, which seriously endangers women's health [25]. Recently, the superiority of molecular therapy in the treatment of BCA has been constantly confirmed and explored [26]. Therefore, there is an urgent need to screen new biomarkers and therapeutic targets to increase the early diagnosis and treatment of BCA.

*ANXA9* is a member of the ANXA group, and it primarily participates in the organization and modulation of membrane/cytoskeleton linkage [27]. Studies have demonstrated that *ANXA9* is strongly upregulated in, and promotes the progression of, different cancers, such as colorectal cancer [16] and gastric cancer [17]. Furthermore, most recently, *ANXA9* was proposed to be closely related to BCA prognosis and is a potential prognostic indicator [18]. However, there was no direct evidence investigating the biological expression and functional role of *ANXA9* in BCA. In the present study, the TIMER database revealed that *ANXA9* was highly expressed in various cancer types, including BCA. The GEPIA database also showed that *ANXA9* was upregulated in BCA samples and differentially expressed in different BCA stages. These results are in agreement with the outcome of a previous report [18]. The UALCAN database showed that *ANXA9* dysregulation is found in BCA cancer patients with different races, ages, clinical stages, cancer subtypes, node metastasis status, and menopause status. Similarly, biological assays further confirmed that *ANXA9* mRNA and ANXA9 protein were notably overexpressed in tumor tissues of BCA patients and also in BCA cells, such as MCF7, SK‐BR‐3, and T‐47D cells. Furthermore, functional assays demonstrated that *ANXA9* depletion could repress BCA cell proliferation, migration, and invasion and facilitate cell apoptosis, suggesting that *ANXA9* functions as an oncogene in BCA. Previously, *ANXA9* overexpression was confirmed to promote cisplatin resistance in ovarian carcinoma cells [28]. In another study, *ANXA9* was shown to be involved in bone metastasis and was related to tumor relapse [29]. These studies further support the outcomes of our study implying the oncogenic function of *ANXA9* in BCA.

Exosomes are small microvesicles ranging from 30 to 100 nm in diameter [30]. They are involved in pathological processes via delivering assorted signal molecules, including mRNAs, miRNAs, nucleic acids, and so on [31]. Exosomes can deliver RNAs from one cell to another to regulate various processes [32]. Accumulating studies have indicated that tumor‐derived exosomes can facilitate tumor growth via regulating cell biological functions [30]. For example, exosomal *LNMAT2* facilitates lymphatic metastasis in bladder cancer [33]. Ovarian cancer cell‐secreted exosomal miR‐205 promotes metastasis by inducing angiogenesis [34]. Exosomal miR‐567 can reverse trastuzumab resistance and repress BCA progression via targeting *ATG5* [35]. Exosomal *MALAT1* accelerates the cell proliferative capability of BCA [36]. In this study, using the STRING database, we first identified that *ANXA9* is closely associated with exosomes. TSG101, CD63, CD81, and CD9 are regarded as biological markers to identify exosomes [37]. *ANXA9* was found to be markedly correlated with *TSG101*, *CD63*, and *CD9*, and was highly expressed in BCA cells treated with exosomes. Thus, we confirmed that *ANXA9* was secreted by BCA tissue‐derived exosomes that function as a tumor promoter in BCA. Although the present study highlights the tumorigenic role of *ANXA9* in BCA progression, we did not examine the molecular mechanisms through which *ANXA9* regulates tumor development; these are under investigation.

Taken together, this study demonstrates that exosome‐derived *ANXA9* is highly expressed in BCA patients and functions as a tumor promoter to facilitate proliferation, migration, and invasion and decrease cell apoptosis. *ANXA9* is also revealed to enhance tumorigenesis *in vivo*. These findings may provide a new therapeutic strategy by targeting ANXA9 for BCA treatment.

## Author contributions statement

CL, YZ and ZQ conceived and designed the experiments. CL and YZ contributed significantly to the experiments. CL, YZ and YJ helped with the experiments and arranging data. YJ and JL performed the data analyses. CL and YZ wrote the draft manuscript. ZQ revised the manuscript. All authors read and approved the final manuscript.

## Ethics statement

All patients provided their written, voluntarily informed consent. All procedures were carried out in accordance with the guidelines outlined in the Helsinki Declaration and this study was approved by the Ethics Committee of Longyan First Affiliated Hospital of Fujian Medical University, Longyan Fujian, PR China. The animal experiments were approved by our hospital's ethics committee for the care and use of animals.

## Data Availability

The datasets generated during and/or analyzed during the current study are available from the corresponding author upon reasonable request.
